# Effect of *Vernonia amygdalina* Del. Leaf Ethanolic Extract on Intoxicated Male Wistar Rats Liver

**DOI:** 10.3390/scipharm85020016

**Published:** 2017-03-23

**Authors:** Maria Immaculata Iwo, Sergia Louisa Sjahlim, Siti Farah Rahmawati

**Affiliations:** 1 Pharmacology Clinical Pharmacy Research Group, School of Pharmacy, Institut Teknologi Bandung, Jl. Ganesha 10, Bandung 40132, Indonesia; sergialouisa@yahoo.co.id (S.L.S.); siti.farah@fa.itb.ac.id (S.F.R.); 2 Biotechnology Biomedical Research Center, Institut Teknologi Bandung, Jl. Ganesha 10, Bandung 40132, Indonesia

**Keywords:** intoxicated rat liver, Rifampicin, INH, *Vernonia amygdalina*

## Abstract

*Vernonia amygdalina* has been shown to have antioxidant activity, and is also expected to have hepatoprotective activity. This study was conducted to study the effect of *V. amygdalina* ethanol extracts on intoxicated rat livers. Fresh leaves were extracted in ethanol, and the hepatoprotective activity was tested on male Wistar rats induced with a combination of isoniazid (INH) and rifampicin. Parameters observed were the activity of the enzyme alanine transferase (ALT), serum albumin levels, liver index, and histopathological of the rat liver. The results showed that 50 and 100 mg/kg rat body weight of *V. amygdalina* ethanol extracts could prevent liver intoxication, starting on day 14. Based on serum albumin concentrations and ALT activity, the high dose extract (100 mg/kg) was more potent as a hepatoprotective agent compared to the extract at a low dose (50 mg/kg). The group of rats treated with a high dose extract showed normal liver index compared to the positive control. Through histology examination, the liver of rats treated with a high dose extract (100 mg/kg) showed minimal liver cell structure damage, and showed similar patterns to the normal rat. Based on these results, it can be concluded that *V. amygdalina* ethanol extracts can be used to protect the liver in a combination of INH and rifampicin as antituberculosis treatment.

## 1. Introduction

The metabolism of drugs and other exogenous compounds mainly takes place in the liver [1]. In addition, most drugs are administered orally and are absorbed from the gastrointestinal tract to the tissues through the liver. Thus, the liver is liable to damage from such drugs and their metabolites [2]. Three of the main drugs responsible for liver damage in patients include tuberculosis (TB) medication, anticancer drugs, and antibiotics.

Tuberculosis remains a major health problem in South-East Asia and is one of the highest causes of morbidity and mortality. It is also the second highest cause of adult deaths after cardiovascular disease, and is the deadliest pathogen out of all the communicable diseases; there are about half a million cases of smear-positive TB in Indonesia. Due to this high rate of incidence of TB, many patients prescribed anti-TB drugs also suffered damage to the liver. According to research by Nurazminah conducted in a hospital in the cities of Jakarta and Cisarua, Indonesia, the percentage of respondents who experienced liver damage caused by anti-TB drugs was 52.2% [3].

In tuberculosis patients, chemotherapy combination drugs are known to cause toxicity of the liver (hepatotoxiticy). Previous studies have shown a 10% elevation of serum hepatocellular enzymes, and a withdrawal of 1–2% of patients who took a standard combination chemotherapy of isoniazid (INH) and rifampicin was seen because of severe hepatotoxicity leading to fulminant hepatitis [4]. Rifampicin is a strong inducer of Cytochromes P450 (CYP450) in the liver, and its combination with INH has been related to an increased risk of hepatotoxicity. Rifampicin induces INH metabolism, therefore increasing hydrazine production (especially in slow acetylators), which can escalate the toxicity [5].

Liver damage can be prevented by using hepatoprotective agents—compounds that mitigate liver damage caused by hepatotoxic agents. Hepatoprotective agents are used in patients suffering from various liver diseases. Several medicinal plants have been known to have hepatoprotective activities, including *Eclipta alba* (Asteraceae), *Glycyrrhizaglabra* (Leguminosae), *Boerhaaviadiffusa* (Nyctaginaceae), *Phyllanthusamarus* (Euphorbiaceae), and *Silybummarianum* (Compositeae). *Vernonia amygdalina* is a shrub that grows throughout tropical Africa and South-East Asia [6]. The plant is generally known as bitter leaf due to the bitterness of its leaves. Based on previous research [7,8,9,10], *V. amygdalina* has been shown to have antioxidant activities by scavenging free radicals, and is also expected to have hepatoprotective activities. This study was conducted to evaluate the hepatoprotective effect of *V. amygdalina* ethanolic extracts in an anti-TB drug-induced animal model.

## 2. Materials and Methods

### 2.1. Plant Material

*Vernonia amygdalina* plants were collected from Salaman Magelang, Central Java, and identified at the Indonesian Institute of Sciences, Research Center for Biology. The identity of the plants collected for this study was determined by the Research Center for Biology, Indonesian Institute of Sciences No 1806/IPH.1.01/If.07/VIII/2016 as *Vernonia amygdalina* Delile, a plant of the Compositae family.

### 2.2. Extraction of *V. amygdalina*

Fresh leaves were air-dried and processed into crude drug. Then, the dried powder was extracted in ethanol 96% (*v*/*v*) using the Soxhlet method. The obtained extract was then concentrated using a rotary evaporator and was then characterized. 

### 2.3. Extract Characterization and Phytochemical Screening

The characteristics of extract were determined according to methods described by Harborne through the following quality parameters: water content, water extractable matter, ethanol extractable matter, extract density, and phytochemical screening [11].

### 2.4. Animals

Male Wistar rats weighing about 200–250 g and ages 10–12 weeks old were obtained from PT Bio Farma, Bandung. The animals were distributed into stainless steel cages (five rats per cage). They were fed a standard diet and water ad libitum. The animals were maintained under standard laboratory conditions: free air circulation, room temperature of about 22–25 °C, and humidity of 55–60%. All rats were acclimatized for 5 days before the experiment. Handling of the animals were guaranteed since the principal investigator has an Animal Welfare Assurance by the office of laboratory animal welfare, NIH, number A5925-01.

### 2.5. Evaluation of Hepatoprotective Activity

A combination of INH and rifampicin were used as inducers of liver damage [5,12], and silymarin was used as the reference hepatoprotective drug. The drug combination was administered orally 1 h before extract administration. The animals were divided into four groups, each group consisting of five animals. Group 1 served as the control group, which was given INH at a dose of 27 mg/kg body weight (bw) and rifampicin at a dose of 54 mg/kg bw. Groups 2 and group 3 were treated with the inductor combination and *V. amygdalina* ethanol extract at a dose of 50 and 100 mg/kg bw, respectively, for 35 days. In Group 4, the animals received the inductor combination and silymarin at a dose of 54 mg/kg bw. The parameters observed were alanine transferase (ALT) activity, serum albumin levels, and the liver index. Rats liver histopathological examination was also performed. The albumin level and ALT were measured using analytical kits obtained from Sclavo Diagnostics International, Sovicille Siena, Italy. The liver index was calculated as follows:

$$Liver\;Index=\frac{liver\;weight}{body\;weight}\times 100$$

After 35 days, the rats were killed through humane procedures using CO_2_ gas, and the livers were then collected for histological assessment. 

### 2.6. Histological Assessment

Livers from rats of different groups were fixated in 10% neutral formalin solution, dehydrated in graded alcohol, and embedded in paraffin. Fine sections were obtained, mounted on glass slides, and counter-stained with hematoxylin–eosin for light microscopic analysis. 

### 2.7. Statistical Analysis

The results are presented as mean ± standard deviation (SD). Analyses were performed using a Student’s *t*-test. *p*-values less than 0.05 were considered statistically significant [13].

## 3. Results and Discussion

The ethanolic extract of the plant showed the characteristics presented in Table 1. 

The extractable matter content can be used to determine the amount of active constituents that were extracted in the solvent used, as well as the solubility of the active constituents of extract in different solvents. Based on the results, the ethanol extract of *V. amygdalina* had poor solubility in water but high solubility in ethanol. Furthermore, from the phytochemical screening (Table 2), it can be observed that the *V. amygdalina* ethanol extract contained alkaloids, flavonoids, saponins, and steroid/triterpenoids.

The serum ALT activities of the animals in each group are presented in Table 3. It can be observed that after seven days of INH and rifampicin administration, the ALT activity of all groups was elevated, indicating that all of the animals’ livers were damaged. The significant increase in ALT activity on day 7 shows that the combination of INH and rifampicin damage the liver. Throughout the experiment, the ALT activity of the control (+) group remained significantly higher (*p* < 0.01) compared to those of the treated groups. This indicates that *V. amygdalina* ethanol extract at the doses used can prevent liver damage. The results also show that the two doses of *V. amygdalina* extract used seemed to have same effect in lowering ALT activity starting after 7 days. Of the two doses, it seems that the higher dose was more potent compared to the lower dose. This is seen from the ALT activity of the group treated with *V. amygdalina* extract at a dose of 100 mg/kg bw, in which after only 14 days the value was no longer significantly different than that at day 0. Meanwhile, the ALT activity of the group treated with 50 mg/kg bw of the extract needed 35 days to return to normal conditions.

In addition to the ALT activity, to determine the effect of the extract on liver function, the albumin concentrations were also analyzed. Albumin is synthesized in the liver; therefore, a decrease in serum albumin concentration could indicate liver damage. The albumin levels of each group are presented in Table 4.

Daily oral administration of INH and rifampicin for seven consecutive days significantly (*p* < 0.01) altered the rats’ liver function, which was characterized by a decrease in serum albumin concentration. However, after 14 days, both extract groups showed no significant changes in albumin concentration compared to the initial condition (D0). In fact, the albumin concentration of these groups increased after the 14th day. These results suggest that both doses of the extract can prevent liver damage.

At end-point of the experiment (D35), the animals were sacrificed and their body weights measured. The livers of the animals were then isolated and weighed to determine the liver index. This parameter was measured to determine the presence of liver enlargement associated with liver damage.

**Figure 1:**
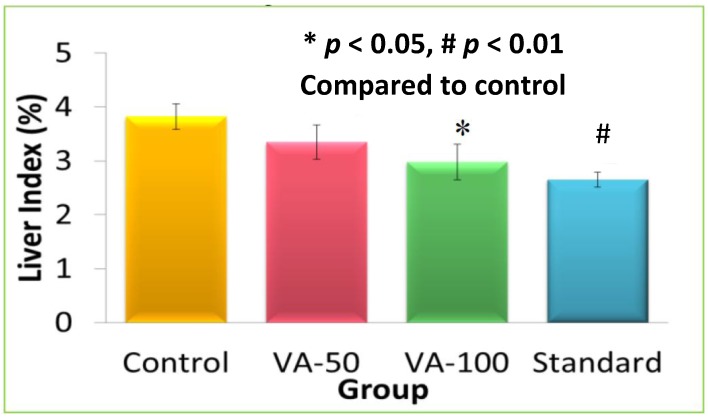
The liver index of the group of rats treated with *V. amygdalina* (VA) extract.

The results of the liver index calculation (Figure 1) showed that the liver index of the group of rats given extract at a dose of 100 mg/kg bw and silymarin were significantly (*p* < 0.05 and *p* < 0.01, respectively) lower compared to the control group. Additionally, the liver index of the 100 mg/kg bw extract group was no different compared to the group given silymarin, suggesting that the extract at a high dose (100 mg/kg bw) has comparable hepatoprotective activity to the reference drug.

**Figure 2:**
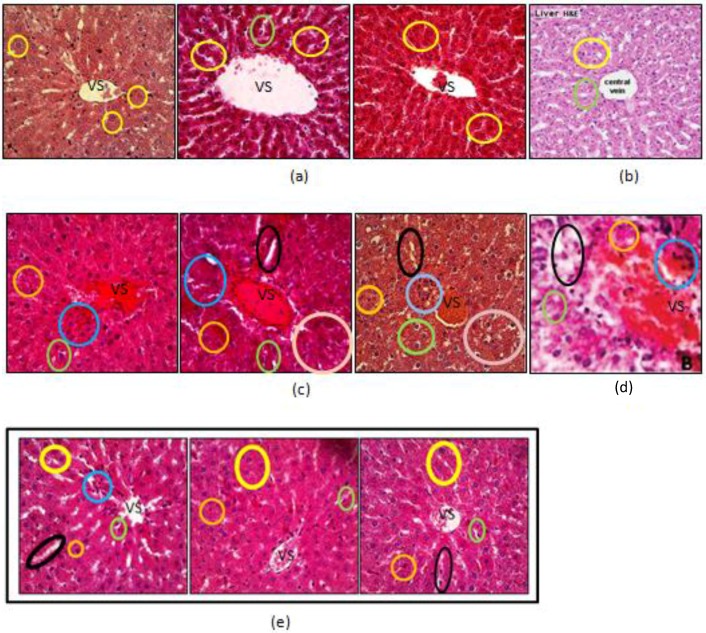

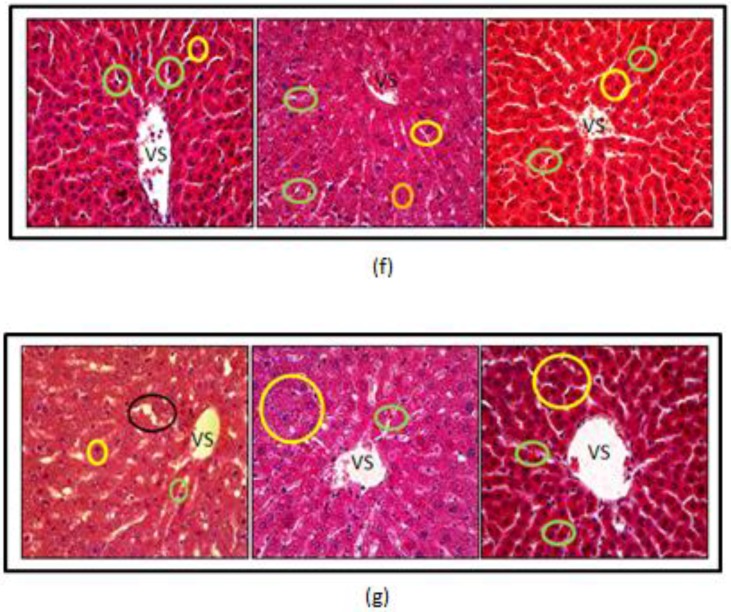
Histopathological sections of rat’s liver. All pictures are magnified 10×10 times. (**a**) Normal control group; (**b**) Histology of the normal liver structure from literature [14]; (**c**) Positive control group; (**d**) Histology of the intoxication liver structure from literature [12]; (**e**) Group treated with extract at a dose of 50 mg/kg bw and (**f**) 100 mg/kg bw; and (**g**) The reference group. VS marks the central vein, yellow marks show normal hepatocytes, green marks show Kupffer cells, black marks show sinusoid dilatation, blue marks show hydropic degeneration, and orange marks show necrotic cells.

The pattern and severity of liver damage of each animal in every group was observed by histological observation. The microscopic cross-sections are presented in Figure 2. 

Histopathological examination of the liver sections from normal rats showed normal parenchymal structure, normal sinusoidal architecture, and no significant lesions were observed. In addition, Kupffer cells—phagocytic cells that engulf pathogens, cell debris, and damaged blood cells—could be found in the lining of the sinusoids. According to Sahai et al. [14] and Junqueira [15], profiles of normal liver histology show hepatocytes with a polyhedral shape and a round nucleus at the center [15,16]. The positive control group showed changes in liver structure that includes inflammation, hydropic degeneration, and necrosis. The parenchyma showed loss of the normal sinusoidal architecture due to numerous foci of lobular inflammation, hepatocyte dropout, and rosette formation. These results were in line with studies that have shown that the special characteristics of liver damage caused by a combination of INH and rifampin are inflammation and cell necrosis [16,17]. According to Mitchell et al., hydropic degeneration occurs due to a disturbance of active transport that prevents the cellular efflux of Na^+^ ions resulting in an increased concentration of intracellular Na^+^ ions. This causes osmosis and an influx of water into the cells, causing the cells to become swollen and have an enlarged nucleus. Granular in the nucleus are also evident [17].

As for the groups administered *V. amygdalina* extract and silymarin, the liver cell damage was lighter than the positive control group. Liver tissue in the group given 50 mg/kg bw of extract showed a microscopic appearance with an orderly arrangement. There was still sinusoidal dilation, but neutrophils could be observed accumulated around the central vein, as well as plenty of Kupffer cells. Liver tissue of the group given 100 mg/kg bw of extract showed lighter liver damage than the 50 mg/kg bw group, but not better than the group given silymarin. The composition of cells in the liver tissue of the high dose group was regularly arranged, and no visible sinusoidal dilation was observed, although there was also a more regular arrangement of hepatocytes. After administration of silymarin, the liver histology showed only slight damage and approached normal circumstances.

## 4. Conclusions

A combination of INH and rifampicin given daily for seven consecutive days can cause significant rat liver intoxication. Ethanolic extracts of *V. amygdalina* leaves at 46 and 92 mg/kg bw (corrected to its water content)—equivalent to 3.58 and 7.17 g of dried leaves—were shown to be able to protect liver intoxication caused by a combination of INH and rifampicin. 

## Figures and Tables

**Table 1:** Characteristics of the extract.

Quality Parameters	Results
Water extractable matter (%)	7.7
Ethanol extractable matter (%)	15.9
Density for 1% extract (g/mL)	0.7
Water content (%)	8

**Table 2:** Phytochemical screening.

Compound	Results
Alkaloids	+
Flavonoids	+
Saponins	+
Tannins	−
Quinones	−
Steroid/Triterpenoids	+

+ = contained; − = not contained.

**Table 3:** Effect of ethanolic extract of *Vernonia amygdalina* (VA) on serum alanine transferase (ALT) activity.

Group	Dose (mg/kg bw)	ALT (U/L)					
		D0	D7	D14	D21	D28	D35
Control (+)	0	43.47 ± 16.24	61.63 ± 17.16 ^x^	80.00 ± 9.09 ^z^	79.57 ± 8.46 ^z^	82.14 ± 6.64 ^z^	84.00 ± 5.29 ^z^
VA extract	50	39.17 ± 10.04	58.14 ± 9.58 ^z^	56.75 ± 10.63 ^c,z^	51.37 ± 3.58 ^c,z^	50.13 ± 5.79 ^c,y^	49.50 ± 4.81 ^c,x^
	100	44.93 ± 15.01	63.01 ± 3.01 ^z^	55.75 ± 4.65 ^c,x^	54.00 ± 5.47 ^c^	49.00 ± 4.24 ^c^	47.37 ± 4.89 ^c^
Reference (silymarin)	54	42.56 ± 12.22	54.56 ± 7.88 ^y^	49.42 ± 3.10 ^c^	50.42 ± 6.39 ^c^	48.86 ± 4.74 ^c^	46.57 ± 5.80 ^c^

Values are expressed as mean ± standard deviation (SD). *n* = 5; Data was analyzed by students *t*-test, where a = *p* < 0.1, b = *p* < 0.05, c = *p* < 0.01 compared to induced control (+) group; x = *p* < 0.1, y = *p* < 0.05, z = *p* < 0.01 compared to D0.

**Table 4:** Effect of ethanolic extract of *V. amygdalina* on serum albumin concentration.

Group	Dose (mg/kg bw)	Albumin Concentration (g/dL)					
		D0	D7	D14	D21	D28	D35
Control (+)	0	3.57 ± 0.35	3.16 ± 0.16 ^y^	2.91 ± 0.35 ^z^	3.00 ± 0.32 ^z^	2.86 ± 0.4 ^z^	2.71 ± 0.31 ^y^
VA Extract	50	3.37 ± 0.47	2.99 ± 0.19 ^a,y^	3.45 ± 0.39 ^b^	3.60 ± 0.38 ^c^	3.69 ± 0.40 ^c^	3.67 ± 0.56 ^c^
	100	3.67 ± 0.49	3.24 ± 0.29 ^x^	3.46 ± 0.40 ^b^	3.70 ± 0.43 ^c^	3.76 ± 0.47 ^c^	3.79 ± 0.47 ^c^
Reference (silymarin)	54	3.43 ± 0.35	3.11 ± 0.23 ^x^	3.46 ± 0.40 ^b^	3.79 ± 0.29 ^c^	3.83 ± 0.80 ^c^	3.87 ± 0.46 ^cx^

Values are expressed as mean ± SD. *n* = 5; Data was analyzed by students *t*-test, where a = *p* < 0.1, b = *p* < 0.05, c = *p* < 0.01 compared to induced control (+) group; x = *p* < 0.1, y = *p* < 0.05, z = *p* < 0.01 compared to D0.
